# Predicting Drug Combination Index and Simulating the Network-Regulation Dynamics by Mathematical Modeling of Drug-Targeted EGFR-ERK Signaling Pathway

**DOI:** 10.1038/srep40752

**Published:** 2017-01-19

**Authors:** Lu Huang, Yuyang Jiang, Yuzong Chen

**Affiliations:** 1 The Ministry-Province Jointly Constructed Base for State Key Lab and Shenzhen Technology and Engineering Lab for Personalized Cancer Diagnostics and Therapeutics Tsinghua University Shenzhen Graduate School, and Shenzhen Kivita Innovative Drug Discovery Institute, Shenzhen, 518055, P.R. China; 2 Institute of Molecular Biology (IMB), Ackermannweg 4, 55128 Mainz, Germany; 3 Department of Pharmacy, and Center for Computational Science and Engineering, National University of Singapore, Blk S16, Level 8, 3 Science Drive 2, 117543 Singapore; 4 State Key Laboratory of Biotherapy, West China Hospital, West China School of Medicine, Sichuan University, Chengdu, China

## Abstract

Synergistic drug combinations enable enhanced therapeutics. Their discovery typically involves the measurement and assessment of drug combination index (CI), which can be facilitated by the development and applications of in-silico CI predictive tools. In this work, we developed and tested the ability of a mathematical model of drug-targeted EGFR-ERK pathway in predicting CIs and in analyzing multiple synergistic drug combinations against observations. Our mathematical model was validated against the literature reported signaling, drug response dynamics, and EGFR-MEK drug combination effect. The predicted CIs and combination therapeutic effects of the EGFR-BRaf, BRaf-MEK, FTI-MEK, and FTI-BRaf inhibitor combinations showed consistent synergism. Our results suggest that existing pathway models may be potentially extended for developing drug-targeted pathway models to predict drug combination CI values, isobolograms, and drug-response surfaces as well as to analyze the dynamics of individual and combinations of drugs. With our model, the efficacy of potential drug combinations can be predicted. Our method complements the developed in-silico methods (e.g. the chemogenomic profile and the statistically-inferenced network models) by predicting drug combination effects from the perspectives of pathway dynamics using experimental or validated molecular kinetic constants, thereby facilitating the collective prediction of drug combination effects in diverse ranges of disease systems.

Synergistic drug combinations have been extensively explored for enhanced therapeutic efficacies^1^,^2^,^3^,^4^,^5^,^6^,^7^,^8^,^9^. In discovering and investigating synergistic drug combinations, the level of synergism is typically measured and quantified by the drug combination index (CI, a quantitative measure of drug combination effects defined in Method Section) such as Chou and Talalay’s CI from experimental dose-response data^1^,^3^,^10^. Based on our literature search study, over 523 papers since 2004 have reported the discovery and optimization of synergistic drug combinations based on the experimentally determined CIs. In-silico tools that can predict CIs without the time-consuming and costly measurement of dose-response data are highly useful for facilitating the discovery of synergistic drug combinations.

Computational methods have been developed for predicting drug combination effects from gene expression profiles of drug-treated samples^11^,^12^,^13^,^14^,^15^ and simulation of drug-targeted signaling^16^,^17^,^18^,^19^,^20^,^21^ and metabolic^22^,^23^,^24^,^25^ pathways. In particular, simulation of drug-targeted pathways is potentially useful for predicting CIs^17^,^26^, as demonstrated by the successful applications of the chemogenomic profile based models^27^,^28^ and the statistically-inferenced network models^29^,^30^ for the prediction of synergistic effects of drug combinations. But the ability of the pathway simulation methods in predicting CIs has not been adequately tested against the observed values of multiple drug combinations targeting multiple target combinations. More tests are needed for determining what the existing mathematical models are capable of and what need to be further improved. These also provide useful knowledge for developing drug or drug combination targeted mathematical models for a number of pathways targeted by drugs and drug combinations (e.g. EGFR-ERK^31^,^32^,^33^,^34^,^35^, apoptosis^36^,^37^, NFκB^16^,^17^, Wnt^19^ and disease-relevant metabolic^22^,^23^,^24^,^25^ pathways).

In this work, we developed and tested a mathematical model of drug and drug combination targeted EGFR-ERK pathway (Fig. 1) based on the ordinary differential equation model of Hornberg^38^. The method for developing this model is provided in the method section. This pathway was selected for two reasons. First, several kinases in this pathway have been targeted by individual inhibitor drugs and drug combinations with available experimental drug response and CI data^39^,^40^,^41^,^42^,^43^,^44^. Secondly, it is one of the pathways with well-established mathematical models^31^,^32^,^33^,^34^,^35^,^38^, ideal for developing and testing drug-targeted pathway mathematical models. The kinase inhibitor drugs included in our mathematical model are EGFR, BRaf and MEK inhibitors, which together with their combinations have been clinically used or tested for the treatment of melanoma, colon, gastric, pancreatic, non-small-cell-lung-cancer (NSCLC) and other cancers^39^,^40^,^41^,^42^,^43^,^44^.

The inhibitory effect of each drug against its target was measured by the percentage reduction of the integrated non-drug-bound target level at different drug concentrations (target dose response curve), and the concentration that induces 50% reduction was taken as the half maximal inhibitory concentration (IC50 value). The integrated non-drug-bound target level refers to the integral of the free target level over the first 2 hours of signaling stimulation. The anti-proliferative effect of each drug or drug combination was measured by the percentage reduction of the integrated phosphorylated ERK (ppERK) level (described below) with respect to the concentration(s) of the drug or drug combination (anti-proliferative dose effect curve)^45^, and the concentration(s) that induce 90% reduction was taken as the half maximal inhibition of growth (GI50 value) of the drug or drug combination (details in the Method Section). The integrated ppERK level refers to the integral of the ppERK level over the first 2 hours of signaling stimulation (the ppERK level typically returns to the basal level <2 hours after HGF stimulation^46^). The anti-proliferative effect of a drug or drug combination was measured by its induced reduction of the integrated ppERK level for the following reason: The level of cell proliferation is linked to DNA synthesis, which is positively associated with the level of the integrated ERK2 activity^47^, while the latter is correlated to the integrated ppERK level. However, a nonlinear correlation between the integrated ERK activity and cell proliferation rates is observed, with significant reduction of proliferation typically occurs when the ppERK level falls below 10% of its peak value^48^,^49^. Therefore, the concentration(s) of a drug or drug combination that induces 90% reduction of the integrated ppERK level was used to measure the GI50 value.

Our mathematical model was validated against the previously observed and simulated effects of the regulation of EGF, PP2A, MKP3 on ERK activities and the regulation of EGF on RasGTP, and its predicted anti-proliferate effects of EGFR, BRaf and MEK inhibitors were compared to the experimental cancer cell-line growth inhibition GI50 values. Additionally, the simulated efficiency of EGFR-MEK combination inhibitors was consistent with experimental results. It was then used to predict the effects of the combinations of EGFR-BRaf and BRaf-MEK inhibitors, and the computed anti-proliferate dose-effect curves were used to derive the drug-combination isobolograms and CIs based on Chou and Talalay’s formula^10^. The predicted CIs were compared to the experimental values for evaluating the performance of the model.

## Results and Discussions

### Model validation against the observed signaling dynamics and the reported simulation results in the absence of a drug

Our mathematical model, described in the Method section, was first validated by comparing the simulated signaling dynamics in the absence of drugs with the literature-reported observations and simulation results. We found that the simulated time-dependent protein concentration profiles are in reasonable agreement with the experimentally determined profiles and the reported simulation results. At 8 nM EGF, the simulated ERK activation peaks within 5 minutes and decays within 2 hours (Supplementary Figure S1), which is consistent with the observed^46^ and the simulated^38^ ERK phosphorylation kinetics. The amount of simulated active RasGTP peaks at ~2 minutes and quickly decays within 10 minutes (Supplementary Figure S2), which is consistent with the observation that active RasGTP level in EGF- treated PC12 cells increases dramatically within 5 minutes and decays steeply within 10 minutes^32^. In simulating the regulation of EGFR-ERK signaling by phosphatases, we found that increasing PP2A level from 30000 to 60000 molecules/cell induces little changes in the peak amplitude but significantly reduces the duration of ERK activation (Supplementary Figure S3), while increasing MKP3 level from 9 × 10^6^ to 1.5 × 10^7^ molecules/cell significantly alters the peak amplitude and substantially reduces the duration of ERK activation (Supplementary Figure S4), which are consistent with the results of a reported simulation study showing that the duration of ERK activation is sensitive only to phosphatase reactions on MEK whereas the amplitude is most sensitive to phosphatase reactions on ERK^50^.

### Model validation against the experimental anti-proliferation activities of individual drugs

Our mathematical model was further validated by the comparison of the predicted GI50 values of individual EGFR, BRaf and MEK inhibitor with the reportedly observed GI50 values of known inhibitors against different cancer cell-lines^51^,^52^. Figure 2 shows the computed anti-proliferative dose-response curves of the EGFR, BRaf and MEK inhibitor respectively, and the computed target inhibitory dose-effect curves of these three inhibitors are shown in Supplementary Figures S5–S7 respectively. From Fig. 2, the computed GI50 values for EGFR, BRaf and MEK inhibitor are 19, 113, 29 nM respectively, which are within 10 fold of the largest experimental GI values against the cancer cell-lines promoted by the EGFR pathway (45 and 53 nM for EGFR inhibitor gefitinib and erlotinib against non-small-cell lung cancer (NSCLC) cell-lines^51^, 1.1 μM for BRaf inhibitor sorafenib against NSCLC cell-lines^50^, and 14–200 nM for MEK inhibitor AZD6244 against thyroid cancer and melanoma cell lines^52^.

**Figure 1:**
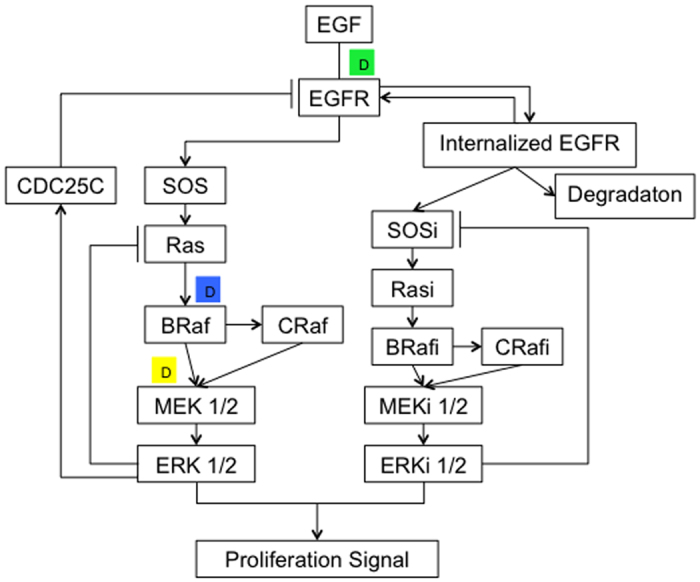
Drug-targeted EGFR-ERK pathway schema in this study. The EGFR, Raf and MEK inhibitor is represented by the small green, blue and yellow colored node with a letter D respectively.

**Figure 2:**
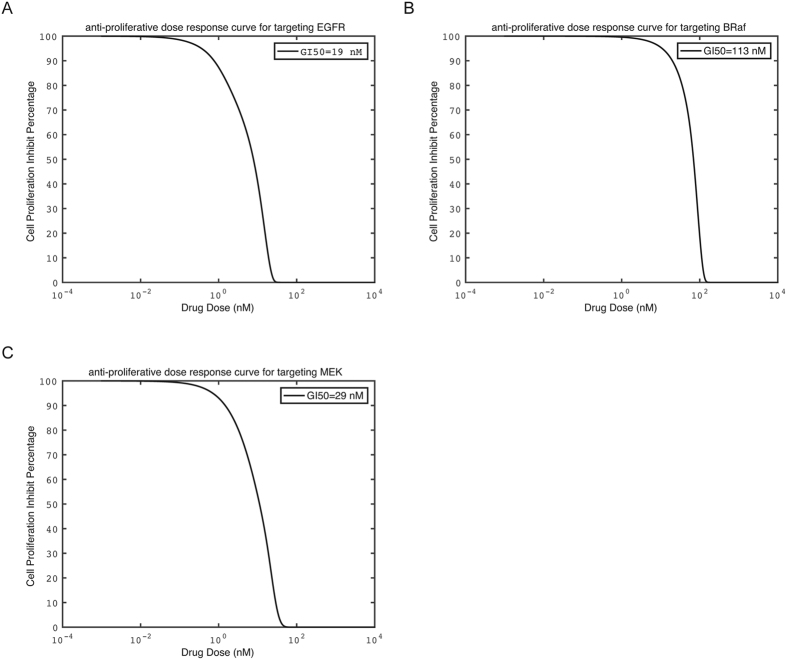
The computed anti-proliferative dose-response curve of individual inhibitors against EGFR-ERK pathway mediated cell growth signaling. (**A**) EGFR inhibitor; (**B**) BRaf inhibitor; (**C**) MEK inhibitor.

The Kd values of the EGFR, BRaf and MEK inhibitors were set such that it gives the median IC50 (10 nM, 30 nM, and 15 nM for EGFR, BRaf, MEK inhibitors respectively) corresponding to the highly potent IC50 values of the drugs frequently used in pharmaceutical research (12.5 ± 7.5 nM against EGFR, 28 ± 3 nM against BRaf, and 15.5 ± 3.5 nM against MEK, details in the Method Section). The predicted GI50 values of the EGFR and MEK inhibitors are substantially smaller than (by 4–6 fold) that of the BRaf inhibitor. This is consistent with the observed behavior of the highly potent inhibitors with the largest experimental GI values (by 5.5–22 fold) against the cancer cell-lines promoted by the EGFR pathway. Analysis of the interaction dynamics of our mathematical model (Fig. 1) suggested that this phenomenon arises from the additional bypass signaling through BRaf-CRaf dimers after BRaf inhibitor binding to BRaf. As a result, increasing activity of the BRaf inhibitor stimulates the bypass signaling mediated through CRaf, and vice versa, leading to an overall less efficient anti-proliferative effect by the BRaf inhibitor than that by the EGFR and MEK inhibitor respectively.

Our mathematical model, was derived from the conventional models^31^,^32^,^33^,^34^,^35^,^38^ and drug-target binding kinetics^34^ with the parameters fitted to the median IC50 values of potent inhibitors. Variation of the IC50 from 5 nM to 50 nM led to comparable degree of variations in the predicted GI50 values (9–95 nM, 19–195 nM and 10–99 nM for EGFR, BRaf and MEK inhibitor respectively). The consistency of the predicted and observed GI values suggests that our mathematical model may have some capability in facilitating the quantitative study of the anti-proliferative activities of the drugs and drug combinations targeting the EGFR pathway.

### Model validation against the experimental anti-proliferation activities of EGFR-MEK inhibitors combination

**Figure 3:**
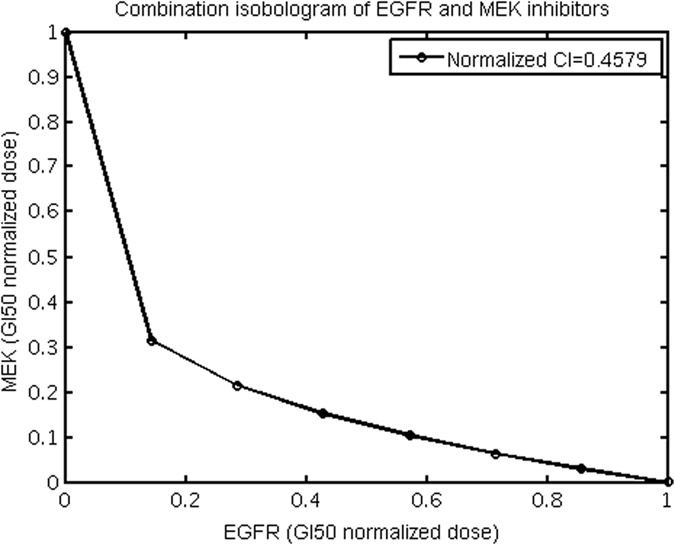
The computed isobologram for the combination of EGFR-MEK inhibitors.

We used our mathematical model to simulate the anti-proliferative effect and to compute the CI values of drug-pair EGFR-MEK inhibitors. As in the cases of drug combination studies^1^,^3^,^10^, the concentration-dependent anti-proliferative effect of drug-pair was measured by an isobologram that displays a curve of the dose-combinations of the two drugs that produce 90% reduction of the integrated ppERK levels within the first 2 hours of signaling stimulation. Figure 3 shows the computed isobologram for the combination of EGFR-MEK inhibitors. The computed CI values for the combinations of EGFR-MEK is 0.46. These are comparable to the experimental values 0.4–0.8 for EGFR-MEK^40^, which suggest that our mathematical model has some level of capability in facilitating the prediction of CI values.

### Prediction of drug combination effects of EGFR-BRaf, BRaf-MEK

**Figure 4:**
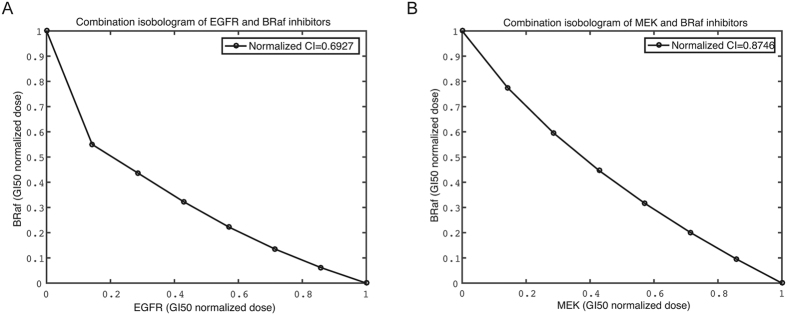
The predicted isobologram for two drug combinations. (**A**) EGFR-BRaf inhibitors; (**B**) BRaf-MEK inhibitors.

BRaf is a commonly mutated oncogene, yet there are no effective therapies exist for BRaf mutant cancers. Mostly because targeting BRaf or MEK kinase in BRaf mutant cancer, respectively, frequently activates other bypass pathways, thus constraining the effectiveness of these inhibitors as single-agents. In our mathematical model, we further predict the combination therapy strategies for BRaf mutant cancers with two drug combination effects, EGFR-BRaf and BRaf-MEK inhibitors, respectively. In modeling of these two drug-pairs, the same set of kinetic parameters for modeling as each individual drug was used. Figure 4 showed the computed isobolograms for the combination of EGFR-BRaf, BRaf-MEK inhibitors. The computed CI values for the combinations of EGFR-BRaf, and BRaf-MEK are 0.69 and 0.87, respectively.

The synergistic effects of these two drug combinations arise from the same type of synergistic mode of action: the complementary action involving positive regulation of a target by targeting two upstream-downstream points of a pathway^8^,^53^,^54^. For the EGFR-BRaf inhibitor combination, the inhibitory activity of the EGFR inhibitor reduces the activation BRaf to complement the inhibitory activity of the BRaf inhibitor. For the BRaf-MEK inhibitor combination, the inhibitory activity of BRaf reduces the activation of MEK to complement the inhibitory activity of MEK inhibitor. These two combinations have largely similar level of synergism as judged by the similar quantities of the computed and observed CI values, which is likely due to the same type of synergistic mode of action.

### Prediction of combination effects of a farnesyltransferase inhibitor combined with a drug targeting BRaf or MEK

Mutated *Ras* genes that produce constitutively active Ras proteins are found to be involved in approximately 30% of human cancers, including several pancreatic and colon carcinomas. Anticancer therapeutic development has been focusing on blockage of hyper-activation of Ras protein, e.g. through farnesyltransferase inhibitors (FTIs) which inhibit the farnesylation of RAS, preventing Ras transform into the functional form that can be attached to the cell-membrane and subsequently transmit signals to the Raf-MEK-ERK (MAPK) signaling pathways, which have a major role in melanoma progression^55^. However, FTIs alone is frequently insufficient for the treatment of cancers. In our mathematical model, we simulated the combination effects of two types of drug combinations, FTI-MEK and FTI-BRaf inhibitors, respectively. FTIs are modeled in our study as drugs inhibiting the transformation of RasGDP to functional RasGTP. The association and dissociation constants *k*_*on*_ and *k*_*off*_ for FTIs are optimized to give IC50 of 10 nM, since most FTIs in pharmaceutical research has less than 10 nM IC50 values^56^,^57^.

**Figure 5:**
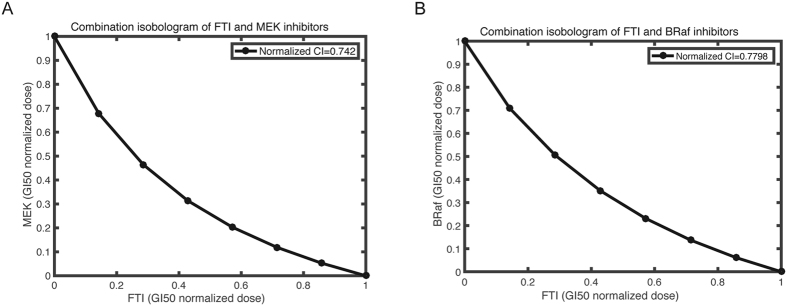
The predicted isobologram for combination treatment of farnesyltransferase inhibitors (FTIs) and drugs. (**A**) FTI-MEK inhibitor; (**B**) FTI-BRaf inhibitor.

Figure 5 showed the computed isobologram for the combination of FTI-MEK inhibitor, FTI-BRaf inhibitor. The computed CI values for the combinations of FTI-MEK inhibitor, and FTI-BRaf inhibitor are 0.74 and 0.78, respectively. The predicted synergism effects of these two drug combinations are consistent with recent literature report that combinations of lonafarnib (a specific FTI) with pan-RAF inhibitor sorafenib yielded additional growth-inhibiting effects than lonafarnib and sorafenib used alone^58^. Moreover, the apoptosis-inducing effect of BMS-214662 (another cytotoxic FTI) has been significantly enhanced through combination treatment with a MEK inhibitor, PD184352, leading to a complete suppression on invasive tumor growth in K562 and CD34 + CML cells^59^. Both our model and experimental studies suggest that the combination treatment of FTI and Raf and MEK-inhibitor may represent an effective alternative for melanoma treatment, and therefore worth further exploration.

## Conclusions

Our drug-targeted EGFR-ERK pathway mathematical model, developed based on existing drug-free models and drug-target interaction kinetics, and validated against a number of published experimental and simulation results, showed fairly good potential in predicting the individual and combination drug effects on the cell-proliferation signaling of the pathway. In particular, the predicted drug combination CI values are comparable to the experimental values for the combination of EGFR-BRaf, EGFR-MEK, BRaf-MEK, FTI-MEK, and FTI-Raf inhibitors^41^, suggesting that existing pathway models may be potentially extended for developing drug-targeted pathway mathematical models to predict drug combination CI values, isobolograms, and drug-response surfaces/curves as well as to analyze the dynamics of individual and combination of drugs. Our model provides a strategy to predict efficacy of these MAPK pathway inhibitors through novel targeted therapy combination approaches.

Further development efforts are needed to explore drug-targeted pathway mathematical models as practical tools for facilitating the discovery of synergistic drug combinations. For instance, some synergistic drug combinations are known to arise from the activities against multiple pathways^8^. Although several multi-pathway models have been developed^60^,^61^,^62^, there is a need to refine existing and develop more multi-pathway models to sufficiently cover the multiple pathways relevant to synergistic drug combinations. Moreover, in some cases, drug effects are influenced by pathway crosstalks^63^,^64^, and drug actions at the multiple sites^65^,^66^, states^67^, conformations^1^, and mutant forms^1^ of the same target. There is a need to add these elements into the existing and newly developed pathway models for extended coverage of therapeutically relevant biological networks and the modes of actions of individual drugs and drug combinations^8^.

## Methods

### Construction of Mathematical Model

Our pathway model schema is illustrated in Fig. 1. We based our model of EGFR signaling on that of Hornberg *et al*., which itself is a refinement of earlier work. The components of drug-target interactions were added to the ordinary differential equation model of Hornberg *et al*.^38^,^68^, with an additional ERK-CDC25C-EGFR feedback loop^44^ and the interactions between BRaf and CRaf^69^ incorporated. The model contains 126 distinct molecular species, and 190 elementary reactions; these reactions are described as a series of ordinary differential equations based on the mass action law. The model is parameterized by 123 kinetic parameters and 126 initial molecular concentrations. A typical enzyme catalyzed reaction in the pathway and the mass-action based rate is shown in the equations below, where the A and B represent species or concentration of species, depending on the context:


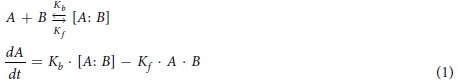


The rate equations are defined by the forward and reverse rate constants *K*_*f*_ and *K*_*b*_ or turnover *K*_*cat*_ used in the published models^31^,^32^,^38^,^70^,^71^,^72^,^73^ or reported from other literatures. The constituent molecular interactions, their kinetic constants, and molecular concentrations are detailed in Supplementary Table S1. Fourth order Runge-Kutta method with adaptive step-size control was used for solving these equations.

### Kinetic Parameters

The types of parameters used in our mathematical model are protein-protein interactions, drug-protein interactions, and catalytic activities. The published simulation studies have shown that most parameters are robust and insensitive to significantly alter the overall pathway behavior^31^,^32^. Apart from the use of the parameters of the published mathematical models, additional parameters were obtained from the literatures based on the widely used assumption that the parameters measured *in vitro* and in some cell lines are generally applicable in most cases. For those protein-protein interactions with unavailable parameters, their parameters were putatively estimated from the known parameters of the relevant interacting domain profile pairs^74^,^75^ or other interacting protein pairs of similar sequences.

### Drug targeting

Drugs targeting each component in the EGFR signaling pathway were simulated as forming complexes with target components based on the mass action law. These inhibitors include the individual EGFR, BRaf, MEK inhibitor or the combination of EGFR-MEK, EGFR-BRaf, and BRaf-MEK inhibitors respectively. For simulating the interaction between each drug and its target, we followed Bairy and Wong^34^ to introduce an extra species, the drug, and two reactions to describe drug-target association and dissociation, with the corresponding kinetic constants *K*_*on*_ and *K*_*off*_ determined such that the *K*_*off*_ takes a value of 0.01 s^−1^, while the *K*_*on*_ was determined such that the drug gives the median IC50 (10 nM, 30 nM, and 15 nM for EGFR, BRaf, MEK inhibitors respectively) corresponding to the highly potent IC50 values of the drugs frequently used in pharmaceutical research (Cetuximab 1 nM, Erlotinib 2 nM, Afatinib 14 nM and Gefitinib 33 nM against EGFR, Sorafenib 25 nM and Vemurafenib 31 nM against BRaf, and AZD6244 12 nM and RDEA119 19 nM against MEK).


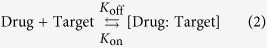


### Definition of IC50 and GI50 values

IC50 is defined as the half maximal inhibitory concentration that induces 50% reduction of integrated non-drug-bound target level. The integrated non-drug-bound target level refers to the integral of the free target level over the first 2 hours of signaling stimulation. For the reasons described in the Introduction Section, the drug concentration-dependent (from 0.001 nM to 10 μM) effects on the ERK-mediated cell-growth signaling process were determined by measuring the percentage of ppERK reduction within the first 2 hours of signaling stimulation. GI50 is defined as the growth inhibition concentration that induces 90% reduction of ppERK within the first 2 hours. Further analysis showed that the variation of the GI50 values from 80% to 99% of ppERK reduction resulted in insignificant changes in the predicted CI values (±0.1), and thus no changes in the qualitative conclusion of the synergistic or antagonistic levels of drug combinations. This indicates the robustness of our drug-binding models in predicting drug combination effects.

### Computation of the combination index and isobolograms for quantitative determination of drug interactions

Quantifying drug interactions in drug combination studies and classifying the interactions into categories of synergy, additivity, or antagonism are of interest to many researchers. Isobologram and combination index (CI) analyses are widely used methods for evaluating drug interactions in combination cancer chemotherapy. The Loewe additivity model has been largely used as a reference model when the combined effect of two drugs is additive. The model can be written as in Equation (3):


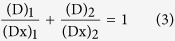


where (*D*)_*1*_ and (*D*)_*2*_ are the respective combination doses of drug 1 and drug 2 that yield an effect of 50% growth inhibition, with (*D*_*x*_)_*1*_ and (*D*_*x*_)_*2*_ being the corresponding single doses for drug 1 and drug 2 that result in the same effect, which is by definition the GI50 of drug 1 and drug 2. When Eq. 3 holds, it can be concluded that the combined effect of the two drugs is additive. Based on Eq. 3, the combination index, defined in Eq. 4, can be used to classify drug interactions as synergistic, additive, or antagonistic.


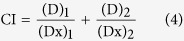



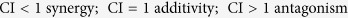


A CI of less than, equal to, and more than 1 indicates synergy, additivity, and antagonism, respectively.

We simulated isobolograms for a pair of drugs with eight equally effective dose combinations for a particular effect level of GI50. GI50 normalized doses of drug 1 and drug 2 that give this effect in combination are plotted as axial points in the isobologram graphs. According to Eq. 4, the isobologram curves are expected to be parallel to the diagonal for additive drug pairs, concave for synergistic drug pairs, and convex for antagonistic drug pairs. We mainly concerned about the qualitative shape of the isobolograms for correctly identifying the drug pair category, and use the smallest CI of the eight drug dose combinations as the CI for this drug pair.

## Additional Information

**How to cite this article**: Huang, L. *et al*. Predicting Drug Combination Index and Simulating the Network-Regulation Dynamics by Mathematical Modeling of Drug-Targeted EGFR-ERK Signaling Pathway. *Sci. Rep.* **7**, 40752; doi: 10.1038/srep40752 (2017).

**Publisher's note:** Springer Nature remains neutral with regard to jurisdictional claims in published maps and institutional affiliations.

## Supplementary Material

Supplementary Tables and Figures
