# Supplementary material for: Predicting Drug Combination Index and Simulating the Network-Regulation Dynamics by Mathematical Modeling of Drug-Targeted EGFR-ERK Signaling Pathway
Source: Sci Rep. 2017 Jan 19;7:40752. doi: 10.1038/srep40752 (PMC5244366; doi:10.1038/srep40752)

Supplementary Tables and Figures for:

Predicting Drug Combination Index and Simulating the Network-Regulation Dynamics by Mathematical Modeling of Drug-Targeted EGFR-ERK Signaling Pathway

Lu Huang [1,2,3], Yuyang Jiang [*,1], Yuzong Chen [*,3,4]

[1]The Ministry-Province Jointly Constructed Base for State Key Lab and Shenzhen Technology and Engineering Lab for Personalized Cancer Diagnostics and Therapeutics Tsinghua University Shenzhen Graduate School, and Shenzhen Kivita Innovative Drug Discovery Institute, Shenzhen, P.R. China, 518055

[2]Institute of Molecular Biology (IMB), Ackermannweg 4, 55128 Mainz, Germany

[3] Department of Pharmacy, and Center for Computational Science and Engineering, National University of Singapore, Blk S16, Level 8, 3 Science Drive 2, Singapore 117543

[4] State Key Laboratory of Biotherapy, West China Hospital, West China School of Medicine, Sichuan University, Chengdu, China.

*Co-corresponding Author: Yuzong Chen. Tel.: 65-6874-6877. Fax: 65-6774-6756. E-mail:

phacyz@nus.edu.sg

Yuyang Jiang. E-mail: jiangyy@sz.tsinghua.edu.cn

Supplementary Table S1: Mathematical models of drug-targeted EGFR-ERK pathway in this study.

Supplementary Table S1a: **Model species initial conditions**

Name	Initial	Input	ERK-PP
EGF	8.00E-09	1	0
EGFR	5.00E+04	0	0
EGF-EGFR	0.00E+00	0	0
(EGF-EGFR)2	0.00E+00	0	0
(EGF-EGFR*)2	0.00E+00	0	0
EGFRi	0.00E+00	0	0
(EGF-EGFR*)2-GAP-Grb2-Prot	0.00E+00	0	0
(EGF-EGFRi*)2	0.00E+00	0	0
Proti	0.00E+00	0	0
EGF-EGFRi	0.00E+00	0	0
(EGF-EGFRi)2	0.00E+00	0	0
Prot	8.10E+04	0	0
GAP	1.20E+04	0	0
(EGF-EGFR*)2-GAP	0.00E+00	0	0
EGFi	0.00E+00	0	0
(EGF-EGFRi*)2-GAP	0.00E+00	0	0
(EGF-EGFRi*)2-GAP-Grb2	0.00E+00	0	0
(EGF-EGFRi*)2-GAP-Grb2-Sos	0.00E+00	0	0
(EGF-EGFRi*)2-GAP-Grb2-Sos-Ras-GDP	0.00E+00	0	0
(EGF-EGFRi*)2-GAP-Grb2-Sos-Ras-GTP	0.00E+00	0	0
Grb2	1.10E+04	0	0
(EGF-EGFR*)2-GAP-Grb2	0.00E+00	0	0
Sos	2.63E+04	0	0
(EGF-EGFR*)2-GAP-Grb2-Sos	0.00E+00	0	0
Ras-GDP	7.20E+04	0	0
(EGF-EGFR*)2-GAP-Grb2-Sos-Ras-GDP	0.00E+00	0	0
Ras-GTP	0.00E+00	0	0
(EGF-EGFR*)2-GAP-Grb2-Sos-Ras-GTP	0.00E+00	0	0
Grb2-Sos	4.00E+04	0	0
Shc	1.01E+05	0	0
(EGF-EGFR*)2-GAP-SHC	0.00E+00	0	0
(EGF-EGFR*)2-GAP-SHC*	0.00E+00	0	0
(EGF-EGFR*)2-GAP-SHC*-Grb2	0.00E+00	0	0
(EGF-EGFR*)2-GAP-SHC*-Grb2-Sos	0.00E+00	0	0
(EGF-EGFR*)2-GAP-SHC*-Grb2-Sos-Ras-GDP	0.00E+00	0	0
(EGF-EGFR*)2-GAP-SHC*-Grb2-Sos-Ras-GTP	0.00E+00	0	0
Shc*-Grb2-Sos	0.00E+00	0	0
Shc*-Grb2	0.00E+00	0	0

Shc*	0.00E+00	0	0
BRaf	4.00E+04	0	0
BRaf-Ras-GTP	0.00E+00	0	0
Ras-GTP*	0.00E+00	0	0
Phosphatase1	4.00E+04	0	0
BRaf*	0.00E+00	0	0
BRaf*-phosphatase1	0.00E+00	0	0
MEK	2.10E+07	0	0
MEK-BRaf*	0.00E+00	0	0
MEK-P	0.00E+00	0	0
MEK-P-BRaf*	0.00E+00	0	0
MEK-PP	0.00E+00	0	0
MEK-PP-phosphatase2	0.00E+00	0	0
phosphatase2	4.00E+04	0	0
MEK-P-phosphatase2	0.00E+00	0	0
ERK	2.21E+07	0	0
ERK-MEK-PP	0.00E+00	0	0
ERK-P	0.00E+00	0	0
ERK-P-MEK-PP	0.00E+00	0	0
ERK-PP	0.00E+00	0	1
phosphatase3	1.00E+07	0	0
ERK-PP-phosphatase3	0.00E+00	0	0
ERK-P-phosphatase3	0.00E+00	0	0
(EGF-EGFRi*)2-GAP-SHC	0.00E+00	0	0
(EGF-EGFRi*)2-GAP-SHC*	0.00E+00	0	0
(EGF-EGFRi*)2-GAP-SHC*-Grb2	0.00E+00	0	0
(EGF-EGFRi*)2-GAP-SHC*-Grb2-Sos	0.00E+00	0	0
(EGF-EGFRi*)2-GAP-SHC*-Grb2-Sos-Ras-GDP	0.00E+00	0	0
(EGF-EGFRi*)2-GAP-SHC*-Grb2-Sos-Ras-GTP	0.00E+00	0	0
Ras-GTPi	0.00E+00	0	0
BRaf-Ras-GTPi	0.00E+00	0	0
Ras-GTPi*	0.00E+00	0	0
BRafi*	0.00E+00	0	0
BRafi*-phosphatase1	0.00E+00	0	0
MEK-BRafi*	0.00E+00	0	0
MEKi-P	0.00E+00	0	0
MEK-P-BRafi*	0.00E+00	0	0
MEKi-PP	0.00E+00	0	0
MEKi-PP-phosphatase2	0.00E+00	0	0
MEKi-P-phosphatase2	0.00E+00	0	0
ERKi-MEKi-PP	0.00E+00	0	0
ERKi-P	0.00E+00	0	0
ERKi-P-MEKi-PP	0.00E+00	0	0
ERKi-PP	0.00E+00	0	1

ERKi-PP-phosphatase3	0.00E+00	0	0
ERKi-P-phosphatase3	0.00E+00	0	0
(EGF-EGFR*)2-GAP-Grb2-Sos-Prot	0.00E+00	0	0
(EGF-EGFR*)2-GAP-Grb2-Sos-Ras-GDP-Prot	0.00E+00	0	0
(EGF-EGFR*)2-GAP-Grb2-Sos-Ras-GTP-Prot	0.00E+00	0	0
(EGF-EGFR*)2-GAP-SHC*-Grb2-Prot	0.00E+00	0	0
(EGF-EGFR*)2-GAP-SHC*-Grb2-Sos-Prot	0.00E+00	0	0
(EGF-EGFR*)2-GAP-SHC*-Grb2-Sos-Ras-GDP-Prot	0.00E+00	0	0
(EGF-EGFR*)2-GAP-SHC*-Grb2-Sos-Ras-GTP-Prot	0.00E+00	0	0
(EGF-EGFR*)2-GAP-Grb2-Sos-ERK-PP	0.00E+00	0	0
(EGF-EGFRi*)2-GAP-Grb2-Sos-ERKi-PP	0.00E+00	0	0
(EGF-EGFR*)2-GAP-SHC*-Grb2-Sos-ERK-PP	0.00E+00	0	0
(EGF-EGFRi*)2-GAP-SHC*-Grb2-Sos-ERKi-PP	0.00E+00	0	0
Sos-ERK-PP	0.00E+00	0	0
Sos-ERKi-PP	0.00E+00	0	0
Sosi	0.00E+00	0	0
GAP-Ras-GTP	0.00E+00	0	0
GAP-Ras-GTPi	0.00E+00	0	0
GAP-Ras-GTP*	0.00E+00	0	0
GAP-Ras-GTPi*	0.00E+00	0	0
Drug1	0.00E+00	1	0
Drug2	0.00E+00	1	0
Drug3	0.00E+00	1	0
MEK-Drug1	0.00E+00	0	0
EGFR-Drug2	0.00E+00	0	0
BRaf-Drug3	0.00E+00	0	0
CDC25C	2.21E+07	0	0
CDC25C*	0.00E+00	0	0
CDC25C-ERK-PP	0.00E+00	0	0
(EGF-EGFR*)2-CDC25C*	0.00E+00	0	0
CRaf	4.00E+03	0	0
BRaf-CRaf-Ras-GTP	0.00E+00	0	0
CRaf*	0.00E+00	0	0
CRaf*-phosphatase1	0.00E+00	0	0
MEK-CRaf*	0.00E+00	0	0
MEK-P-CRaf*	0.00E+00	0	0
BRaf-CRaf-Ras-GTPi	0.00E+00	0	0
CRafi*	0.00E+00	0	0
CRafi*-phosphatase1	0.00E+00	0	0
MEK-CRafi*	0.00E+00	0	0
MEK-P-CRafi*	0.00E+00	0	0
BRaf-Drug3-Ras-GTP	0.00E+00	0	0
BRaf*-Drug3	0.00E+00	0	0
BRaf-Drug3-CRaf-Ras-GTP	0.00E+00	0	0

Supplementary Table S1b: Model parameters

Name	Value
k0	0.00E+00
kd0	0.00E+00
k1	3.00E+07
kd1	3.84E-03
k10b	5.43E-02
kd10	1.10E-02
k2	1.66E-05
kd2	1.00E-01
k3	1.00E+00
kd3	1.00E-02
k4	1.73E-07
kd4	1.66E-03
kd5	1.48E-02
k5	0.00E+00
k6	5.00E-04
kd6	5.00E-03
k8	1.66E-06
kd8	2.00E-01
k13	2.17E+00
kd13	0.00E+00
k15	1.00E+04
kd15	0.00E+00
k16	1.66E-05
kd16	0.00E+00
k17	1.66E-05
kd17	6.00E-02
k18	2.50E-05
kd18	1.30E+00
k19	1.66E-07
kd19	5.00E-01
k20	3.50E-06
kd20	4.00E-01
k21	3.66E-07
kd21	2.30E-02
k22	3.50E-05
kd22	1.00E-01
k23	6.00E+00
kd23	6.00E-02
kd24	5.50E-01
k25	1.66E-05
kd25	2.14E-02

k28	1.66E-06
kd28	5.30E-03
k29	1.17E-06
kd29	1.00E+00
kd32	1.00E-01
k32	4.00E-07
kd33	2.00E-01
k33	3.50E-05
kd34	3.00E-02
k34	7.50E-06
kd35	1.50E-03
k35	7.50E-06
k36	5.00E-03
kd36	0.00E+00
kd37	3.00E-01
k37	1.50E-06
k40	5.00E-05
kd40	6.40E-02
k41	5.00E-05
kd41	4.29E-02
k42	1.18E-04
kd42	2.00E-01
kd43	1.00E+00
k43	0.00E+00
kd44	1.83E-02
kd45	3.50E+00
k45	0.00E+00
kd47	2.90E+00
k47	0.00E+00
k48	2.38E-05
kd48	8.00E-01
kd49	5.80E-02
k49	0.00E+00
k50	4.50E-07
kd50	5.00E-01
kd52	3.30E-02
kd53	1.60E+01
k53	0.00E+00
kd55	5.70E+00
k55	0.00E+00
kd56	6.00E-01
k56	2.35E-05
kd57	2.46E-01
k57	0.00E+00

k58	8.33E-06
kd58	5.00E-01
k52	8.91E-05
k44	1.96E-05
k60	5.50E-03
kd60	0.00E+00
k61	6.70E-04
kd61	0.00E+00
kd63	2.75E-01
k63	0.00E+00
k126	1.66E-07
kd126	2.00E+00
kd127	1.00E-04
k127	0.00E+00
k200	2.17E+00
kd214	1.81E-04
kd222	1.97E-04
kd224	8.25E-05
kd226	3.01E-05
kd231	3.00E-05
kd230	5.43E-05
k300	1.00E-07
k401	8.5114E+05
k402	2.3392E+09
k403	1.9055E+05
kd401	1.00E-02
kd402	1.00E-02
kd403	1.00E-02
kadd1	8.91E-10
kadd2	1.60E+00
kadd3	8.33E-11
kadd4	2.46E-02
kdadd1	1.83E-02
kdadd2	1.60E+01
kdadd3	5.00E-01
kdadd4	2.46E-01
k288	1.66E-08
kd288	5.30E-03

Supplementary Table S1c: Model reactions

Reactant1	Reactant2	Product	kForward	kReverse
MEK	Drug1	MEK-Drug1	k401	kd401
EGFR	Drug2	EGFR-Drug2	k402	kd402
BRaf	Drug3	BRaf-Drug3	k403	kd403
GAP	0	0	kd214	k200
Grb2	0	0	kd222	k200
Sos	0	0	kd224	k200
Ras-GDP	0	0	kd226	k200
Shc	0	0	kd231	k200
Grb2-Sos	0	0	kd230	k200
EGFRi	0	0	k60	kd60
EGFi	0	0	k61	kd61
(EGF-EGFRi*)2	0	0	k60	kd60
(EGF-EGFRi*)2-GAP	0	0	k60	kd60
(EGF-EGFRi*)2-GAP-Grb2	0	0	k60	kd60
(EGF-EGFRi*)2-GAP-Grb2-Sos	0	0	k60	kd60
(EGF-EGFRi*)2-GAP-Grb2-Sos-Ras-GDP	0	0	k60	kd60
(EGF-EGFRi*)2-GAP-Grb2-Sos-Ras-GTP	0	0	k60	kd60
(EGF-EGFRi*)2-GAP-SHC	0	0	k60	kd60
(EGF-EGFRi*)2-GAP-SHC*	0	0	k60	kd60
(EGF-EGFRi*)2-GAP-SHC*-Grb2	0	0	k60	kd60
(EGF-EGFRi*)2-GAP-SHC*-Grb2-Sos	0	0	k60	kd60
(EGF-EGFRi*)2-GAP-SHC*-Grb2-Sos-Ras-GDP	0	0	k60	kd60
(EGF-EGFRi*)2-GAP-SHC*-Grb2-Sos-Ras-GTP	0	0	k60	kd60
ERK-PP	phosphatase3	ERK-PP-phosphatase3	k56	kd56
ERK-P	phosphatase3	ERK-PP-phosphatase3	k57	kd57
phosphatase3	ERK-P	ERK-P-phosphatase3	k58	kd58
ERK	phosphatase3	ERK-P-	k57	kd57

		phosphatase3		
ERKi-PP	phosphatase3	ERKi-PP-phosphatase3	k56	kd56
ERKi-P	phosphatase3	ERKi-PP-phosphatase3	k57	kd57
phosphatase3	ERKi-P	ERKi-P-phosphatase3	k58	kd58
ERK	phosphatase3	ERKi-P-phosphatase3	k57	kd57
ERK	MEK-PP	ERK-MEK-PP	k52	kd44
MEK-PP	ERK-P	ERK-MEK-PP	k53	kd53
MEK-PP	ERK-P	ERK-P-MEK-PP	k52	kd44
ERK-PP	MEK-PP	ERK-P-MEK-PP	k55	kd55
ERK	MEKi-PP	ERKi-MEKi-PP	k52	kd44
ERKi-P	MEKi-PP	ERKi-MEKi-PP	k53	kd53
MEKi-PP	ERKi-P	ERKi-P-MEKi-PP	k52	kd44
ERKi-PP	MEKi-PP	ERKi-P-MEKi-PP	k55	kd55
(EGF-EGFR*)2-GAP-Grb2	Prot	(EGF-EGFR*)2-GAP-Grb2-Prot	k4	kd4
(EGF-EGFRi*)2-GAP-Grb2	Proti	(EGF-EGFR*)2-GAP-Grb2-Prot	k5	kd5
EGFR	0	EGFRi	k6	kd6
(EGF-EGFR*)2	0	(EGF-EGFRi*)2	k6	kd6
(EGF-EGFR*)2-GAP-Grb2	0	(EGF-EGFRi*)2-GAP-Grb2	k6	kd6
Proti	0	Prot	k15	kd15
(EGF-EGFR*)2-GAP	0	(EGF-EGFRi*)2-GAP	k6	kd6
(EGF-EGFR*)2-GAP-SHC	0	(EGF-EGFRi*)2-GAP-SHC	k6	kd6
(EGF-EGFR*)2-GAP-SHC*	0	(EGF-EGFRi*)2-GAP-SHC*	k6	kd6
(EGF-EGFR*)2-GAP-Grb2-Sos	0	(EGF-EGFRi*)2-GAP-Grb2-Sos	k6	kd6
(EGF-EGFR*)2-GAP-Grb2-Sos	Prot	(EGF-EGFR*)2-GAP-Grb2-Sos-Prot	k4	kd4
Proti	(EGF-EGFRi*)2-GAP-Grb2-Sos	(EGF-EGFR*)2-GAP-Grb2-Sos-Prot	k5	kd5
(EGF-EGFR*)2-GAP-Grb2-Sos-Ras-GDP	0	(EGF-EGFRi*)2-GAP-Grb2-Sos-Ras-GDP	k6	kd6
(EGF-EGFR*)2-GAP-Grb2-Sos-Ras-GDP	Prot	(EGF-EGFR*)2-GAP-Grb2-Sos-	k4	kd4

		Ras-GDP-Prot		
Proti	(EGF-EGFRi*)2-GAP-Grb2-Sos-Ras-GDP	(EGF-EGFR*)2-GAP-Grb2-Sos-Ras-GDP-Prot	k5	kd5
(EGF-EGFR*)2-GAP-Grb2-Sos-Ras-GTP	0	(EGF-EGFRi*)2-GAP-Grb2-Sos-Ras-GTP	k6	kd6
(EGF-EGFR*)2-GAP-Grb2-Sos-Ras-GTP	Prot	(EGF-EGFR*)2-GAP-Grb2-Sos-Ras-GTP-Prot	k4	kd4
Proti	(EGF-EGFRi*)2-GAP-Grb2-Sos-Ras-GTP	(EGF-EGFR*)2-GAP-Grb2-Sos-Ras-GTP-Prot	k5	kd5
(EGF-EGFR*)2-GAP-SHC*-Grb2	0	(EGF-EGFRi*)2-GAP-SHC*-Grb2	k6	kd6
(EGF-EGFR*)2-GAP-SHC*-Grb2	Prot	(EGF-EGFR*)2-GAP-SHC*-Grb2-Prot	k4	kd4
Proti	(EGF-EGFRi*)2-GAP-SHC*-Grb2	(EGF-EGFR*)2-GAP-SHC*-Grb2-Prot	k5	kd5
(EGF-EGFR*)2-GAP-SHC*-Grb2-Sos	0	(EGF-EGFRi*)2-GAP-SHC*-Grb2-Sos	k6	kd6
(EGF-EGFR*)2-GAP-SHC*-Grb2-Sos	Prot	(EGF-EGFR*)2-GAP-SHC*-Grb2-Sos-Prot	k4	kd4
Proti	(EGF-EGFRi*)2-GAP-SHC*-Grb2-Sos	(EGF-EGFR*)2-GAP-SHC*-Grb2-Sos-Prot	k5	kd5
(EGF-EGFR*)2-GAP-SHC*-Grb2-Sos-Ras-GDP	0	(EGF-EGFRi*)2-GAP-SHC*-Grb2-Sos-Ras-GDP	k6	kd6
(EGF-EGFR*)2-GAP-SHC*-Grb2-Sos-Ras-GDP	Prot	(EGF-EGFR*)2-GAP-SHC*-Grb2-Sos-Ras-GDP-Prot	k4	kd4
Proti	(EGF-EGFRi*)2-GAP-SHC*-Grb2-Sos-Ras-GDP	(EGF-EGFR*)2-GAP-SHC*-Grb2-Sos-Ras-GDP-Prot	k5	kd5

(EGF-EGFR*)2-GAP-SHC*-Grb2-Sos-Ras-GTP	0	(EGF-EGFRi*)2-GAP-SHC*-Grb2-Sos-Ras-GTP	k6	kd6
(EGF-EGFR*)2-GAP-SHC*-Grb2-Sos-Ras-GTP	Prot	(EGF-EGFR*)2-GAP-SHC*-Grb2-Sos-Ras-GTP-Prot	k4	kd4
(EGF-EGFRi*)2-GAP-SHC*-Grb2-Sos-Ras-GTP	Proti	(EGF-EGFR*)2-GAP-SHC*-Grb2-Sos-Ras-GTP-Prot	k5	kd5
MEK-PP	phosphatase2	MEK-PP-phosphatase2	k48	kd48
MEK-P	phosphatase2	MEK-PP-phosphatase2	k49	kd49
phosphatase2	MEK-P	MEK-P-phosphatase2	k50	kd50
MEK	phosphatase2	MEK-P-phosphatase2	k49	kd49
MEKi-PP	phosphatase2	MEKi-PP-phosphatase2	k48	kd48
MEKi-P	phosphatase2	MEKi-PP-phosphatase2	k49	kd49
phosphatase2	MEKi-P	MEKi-P-phosphatase2	k50	kd50
MEK	phosphatase2	MEKi-P-phosphatase2	k49	kd49
ERK-PP	(EGF-EGFR*)2-GAP-Grb2-Sos	(EGF-EGFR*)2-GAP-Grb2-Sos-ERK-PP	k126	kd126
ERKi-PP	(EGF-EGFRi*)2-GAP-Grb2-Sos	(EGF-EGFRi*)2-GAP-Grb2-Sos-ERKi-PP	k126	kd126
ERK-PP	(EGF-EGFR*)2-GAP-SHC*-Grb2-Sos	(EGF-EGFR*)2-GAP-SHC*-Grb2-Sos-ERK-PP	k126	kd126
ERKi-PP	(EGF-EGFRi*)2-GAP-SHC*-Grb2-Sos	(EGF-EGFRi*)2-GAP-SHC*-Grb2-Sos-ERKi-PP	k126	kd126
ERK-PP	Sos	Sos-ERK-PP	k126	kd126
ERKi-PP	Sos	Sos-ERKi-PP	k126	kd126
ERK-PP	0	(EGF-EGFR*)2-GAP-Grb2-Sos-ERK-PP	k127	kd127
ERK-PP	0	(EGF-EGFR*)2-	k127	kd127

		GAP-SHC*-Grb2-Sos-ERK-PP		
ERK-PP	Sosi	Sos-ERK-PP	k127	kd127
ERKi-PP	0	(EGF-EGFRi*)2-GAP-Grb2-Sos-ERKi-PP	k127	kd127
ERKi-PP	0	(EGF-EGFRi*)2-GAP-SHC*-Grb2-Sos-ERKi-PP	k127	kd127
ERKi-PP	Sosi	Sos-ERKi-PP	k127	kd127
Phosphatase1	BRaf*	BRaf*-phosphatase1	k42	kd42
BRaf	Phosphatase1	BRaf*-phosphatase1	k43	kd43
Phosphatase1	BRafi*	BRafi*-phosphatase1	k42	kd42
BRaf	Phosphatase1	BRafi*-phosphatase1	k43	kd43
MEK	BRaf*	MEK-BRaf*	k44	kd52
MEK-P	BRaf*	MEK-BRaf*	k45	kd45
MEK-P	BRaf*	MEK-P-BRaf*	k44	kd52
MEK-PP	BRaf*	MEK-P-BRaf*	k47	kd47
MEK	BRafi*	MEK-BRafi*	k44	kd52
MEKi-P	BRafi*	MEK-BRafi*	k45	kd45
BRafi*	MEKi-P	MEK-P-BRafi*	k44	kd52
BRafi*	MEKi-PP	MEK-P-BRafi*	k47	kd47
Phosphatase1	CRaf*	CRaf*-phosphatase1	k42	kd42
CRaf	Phosphatase1	CRaf*-phosphatase1	k43	kd43
Phosphatase1	CRafi*	CRafi*-phosphatase1	k42	kd42
CRaf	Phosphatase1	CRafi*-phosphatase1	k43	kd43
MEK	CRaf*	MEK-CRaf*	k44	kd52
MEK-P	CRaf*	MEK-CRaf*	k45	kd45
MEK-P	CRaf*	MEK-P-CRaf*	k44	kd52
MEK-PP	CRaf*	MEK-P-CRaf*	k47	kd47
MEK	CRafi*	MEK-CRafi*	k44	kd52
MEKi-P	CRafi*	MEK-CRafi*	k45	kd45
CRafi*	MEKi-P	MEK-P-CRafi*	k44	kd52
CRafi*	MEKi-PP	MEK-P-CRafi*	k47	kd47
Ras-GTP	BRaf	BRaf-Ras-GTP	k28	kd28
Ras-GTP*	BRaf*	BRaf-Ras-GTP	k29	kd29

BRaf-Ras-GTP	CRaf	BRaf-CRaf-Ras-GTP	k28	kd28
BRaf-Ras-GTP	CRaf*	BRaf-CRaf-Ras-GTP	k29	kd29
Ras-GTPi	BRaf	BRaf-Ras-GTPi	k28	kd28
Ras-GTPi*	BRafi*	BRaf-Ras-GTPi	k29	kd29
BRaf-Ras-GTPi	CRaf	BRaf-CRaf-Ras-GTPi	k28	kd28
BRaf-Ras-GTPi	CRafi*	BRaf-CRaf-Ras-GTPi	k29	kd29
Ras-GTP	BRaf-Drug3	BRaf-Drug3-Ras-GTP	k28	kd28
Ras-GTP*	BRaf*-Drug3	BRaf-Drug3-Ras-GTP	k29	kd29
BRaf-Drug3-Ras-GTP	CRaf	BRaf-Drug3-CRaf-Ras-GTP	k288	kd288
BRaf-Ras-GTP	CRaf*	BRaf-Drug3-CRaf-Ras-GTP	k29	kd29
Ras-GDP	(EGF-EGFR*)2-GAP-Grb2-Sos	(EGF-EGFR*)2-GAP-Grb2-Sos-Ras-GDP	k18	kd18
Ras-GTP	(EGF-EGFR*)2-GAP-Grb2-Sos	(EGF-EGFR*)2-GAP-Grb2-Sos-Ras-GDP	k19	kd19
Ras-GDP	(EGF-EGFR*)2-GAP-SHC*-Grb2-Sos	(EGF-EGFR*)2-GAP-SHC*-Grb2-Sos-Ras-GDP	k18	kd18
(EGF-EGFR*)2-GAP-SHC*-Grb2-Sos	Ras-GTP	(EGF-EGFR*)2-GAP-SHC*-Grb2-Sos-Ras-GDP	k19	kd19
Ras-GDP	(EGF-EGFRi*)2-GAP-Grb2-Sos	(EGF-EGFRi*)2-GAP-Grb2-Sos-Ras-GDP	k18	kd18
Ras-GTPi	(EGF-EGFRi*)2-GAP-Grb2-Sos	(EGF-EGFRi*)2-GAP-Grb2-Sos-Ras-GDP	k19	kd19
Ras-GDP	(EGF-EGFRi*)2-GAP-SHC*-Grb2-Sos	(EGF-EGFRi*)2-GAP-SHC*-Grb2-Sos-Ras-GDP	k18	kd18
(EGF-EGFRi*)2-GAP-SHC*-Grb2-Sos	Ras-GTPi	(EGF-EGFRi*)2-GAP-SHC*-Grb2-Sos-Ras-GDP	k19	kd19
(EGF-EGFRi*)2	GAP	(EGF-EGFRi*)2-	k8	kd8

		GAP		
(EGF-EGFR*)2-GAP-Grb2-Sos	Ras-GTP*	(EGF-EGFR*)2-GAP-Grb2-Sos-Ras-GTP	k20	kd20
(EGF-EGFR*)2-GAP-Grb2-Sos	Ras-GDP	(EGF-EGFR*)2-GAP-Grb2-Sos-Ras-GTP	k21	kd21
(EGF-EGFR*)2-GAP-SHC*-Grb2-Sos	Ras-GTP*	(EGF-EGFR*)2-GAP-SHC*-Grb2-Sos-Ras-GTP	k20	kd20
(EGF-EGFR*)2-GAP-SHC*-Grb2-Sos	Ras-GDP	(EGF-EGFR*)2-GAP-SHC*-Grb2-Sos-Ras-GTP	k21	kd21
Ras-GTPi*	(EGF-EGFRi*)2-GAP-Grb2-Sos	(EGF-EGFRi*)2-GAP-Grb2-Sos-Ras-GTP	k20	kd20
(EGF-EGFRi*)2-GAP-Grb2-Sos	Ras-GDP	(EGF-EGFRi*)2-GAP-Grb2-Sos-Ras-GTP	k21	kd21
Ras-GTPi*	(EGF-EGFRi*)2-GAP-SHC*-Grb2-Sos	(EGF-EGFRi*)2-GAP-SHC*-Grb2-Sos-Ras-GTP	k20	kd20
(EGF-EGFRi*)2-GAP-SHC*-Grb2-Sos	Ras-GDP	(EGF-EGFRi*)2-GAP-SHC*-Grb2-Sos-Ras-GTP	k21	kd21
EGF	EGFR	EGF-EGFR	k1	kd1
EGF-EGFR	EGF-EGFR	(EGF-EGFR)2	k2	kd2
(EGF-EGFR)2	0	(EGF-EGFR*)2	k3	kd3
CDC25C	ERK-PP	CDC25C-ERK-PP	kadd1	kdadd1
CDC25C*	ERK-PP	CDC25C-ERK-PP	kadd2	kdadd2
(EGF-EGFR*)2	CDC25C*	(EGF-EGFR*)2-CDC25C*	kadd3	kdadd3
(EGF-EGFR)2	CDC25C*	(EGF-EGFR*)2-CDC25C*	kadd4	kdadd4
EGFRi	EGFi	EGF-EGFRi	k10b	kd10
EGF-EGFRi	EGF-EGFRi	(EGF-EGFRi)2	k2	kd2
(EGF-EGFRi)2	0	(EGF-EGFRi*)2	k3	kd3
0	0	EGFR	k13	kd13
(EGF-EGFR*)2	GAP	(EGF-EGFR*)2-GAP	k8	kd8
Grb2	(EGF-EGFR*)2-GAP	(EGF-EGFR*)2-GAP-Grb2	k16	kd63
Sos	(EGF-EGFR*)2-GAP-	(EGF-EGFR*)2-GAP-Grb2-Sos	k17	kd17

	Grb2			
Shc	(EGF-EGFR*)2-GAP	(EGF-EGFR*)2-GAP-SHC	k22	kd22
(EGF-EGFR*)2-GAP-SHC	0	(EGF-EGFR*)2-GAP-SHC*	k23	kd23
Grb2	(EGF-EGFR*)2-GAP-SHC*	(EGF-EGFR*)2-GAP-SHC*-Grb2	k16	kd24
Sos	(EGF-EGFR*)2-GAP-SHC*-Grb2	(EGF-EGFR*)2-GAP-SHC*-Grb2-Sos	k25	kd25
(EGF-EGFR*)2-GAP	Shc*-Grb2-Sos	(EGF-EGFR*)2-GAP-SHC*-Grb2-Sos	k32	kd32
Shc*	Grb2-Sos	Shc*-Grb2-Sos	k33	kd33
(EGF-EGFR*)2-GAP	Grb2-Sos	(EGF-EGFR*)2-GAP-Grb2-Sos	k34	kd34
Sos	Grb2	Grb2-Sos	k35	kd35
Shc*	0	Shc	k36	kd36
(EGF-EGFR*)2-GAP	Shc*	(EGF-EGFR*)2-GAP-SHC*	k37	kd37
Grb2	Shc*	Shc*-Grb2	k16	kd24
(EGF-EGFR*)2-GAP	Shc*-Grb2	(EGF-EGFR*)2-GAP-SHC*-Grb2	k37	kd37
Sos	Shc*-Grb2	Shc*-Grb2-Sos	k40	kd40
Grb2-Sos	(EGF-EGFR*)2-GAP-SHC*	(EGF-EGFR*)2-GAP-SHC*-Grb2-Sos	k41	kd41
(EGF-EGFRi*)2-GAP	Grb2	(EGF-EGFRi*)2-GAP-Grb2	k16	kd63
Sos	(EGF-EGFRi*)2-GAP-Grb2	(EGF-EGFRi*)2-GAP-Grb2-Sos	k17	kd17
Shc	(EGF-EGFRi*)2-GAP	(EGF-EGFRi*)2-GAP-SHC	k22	kd22
(EGF-EGFRi*)2-GAP-SHC	0	(EGF-EGFRi*)2-GAP-SHC*	k23	kd23
Grb2	(EGF-EGFRi*)2-GAP-SHC*	(EGF-EGFRi*)2-GAP-SHC*-Grb2	k16	kd24
Sos	(EGF-EGFRi*)2-GAP-SHC*-Grb2	(EGF-EGFRi*)2-GAP-SHC*-Grb2-Sos	k25	kd25
(EGF-EGFRi*)2-GAP	Shc*-Grb2-Sos	(EGF-EGFRi*)2-	k32	kd32

		GAP-SHC*-Grb2-Sos		
(EGF-EGFRi*)2-GAP	Grb2-Sos	(EGF-EGFRi*)2-GAP-Grb2-Sos	k34	kd34
(EGF-EGFRi*)2-GAP	Shc*	(EGF-EGFRi*)2-GAP-SHC*	k37	kd37
(EGF-EGFRi*)2-GAP	Shc*-Grb2	(EGF-EGFRi*)2-GAP-SHC*-Grb2	k37	kd37
Grb2-Sos	(EGF-EGFRi*)2-GAP-SHC*	(EGF-EGFRi*)2-GAP-SHC*-Grb2-Sos	k41	kd41
GAP	Ras-GTP	GAP-Ras-GTP	k300	kd20
GAP	Ras-GDP	GAP-Ras-GTP	0	kd21
GAP	Ras-GTPi	GAP-Ras-GTPi	k300	kd20
GAP	Ras-GDP	GAP-Ras-GTPi	0	kd21
GAP	Ras-GTP*	GAP-Ras-GTP*	k300	kd20
GAP	Ras-GDP	GAP-Ras-GTP*	0	kd21
GAP	Ras-GTPi*	GAP-Ras-GTPi*	k300	kd20
GAP	Ras-GDP	GAP-Ras-GTPi*	0	kd21
Sosi	Ras-GDP	Sos	k300	0

Supplementary Figure S1: The simulated time-dependent ERK activation dynamics of the drug-free EGFR-ERK pathway at 8nM EGF

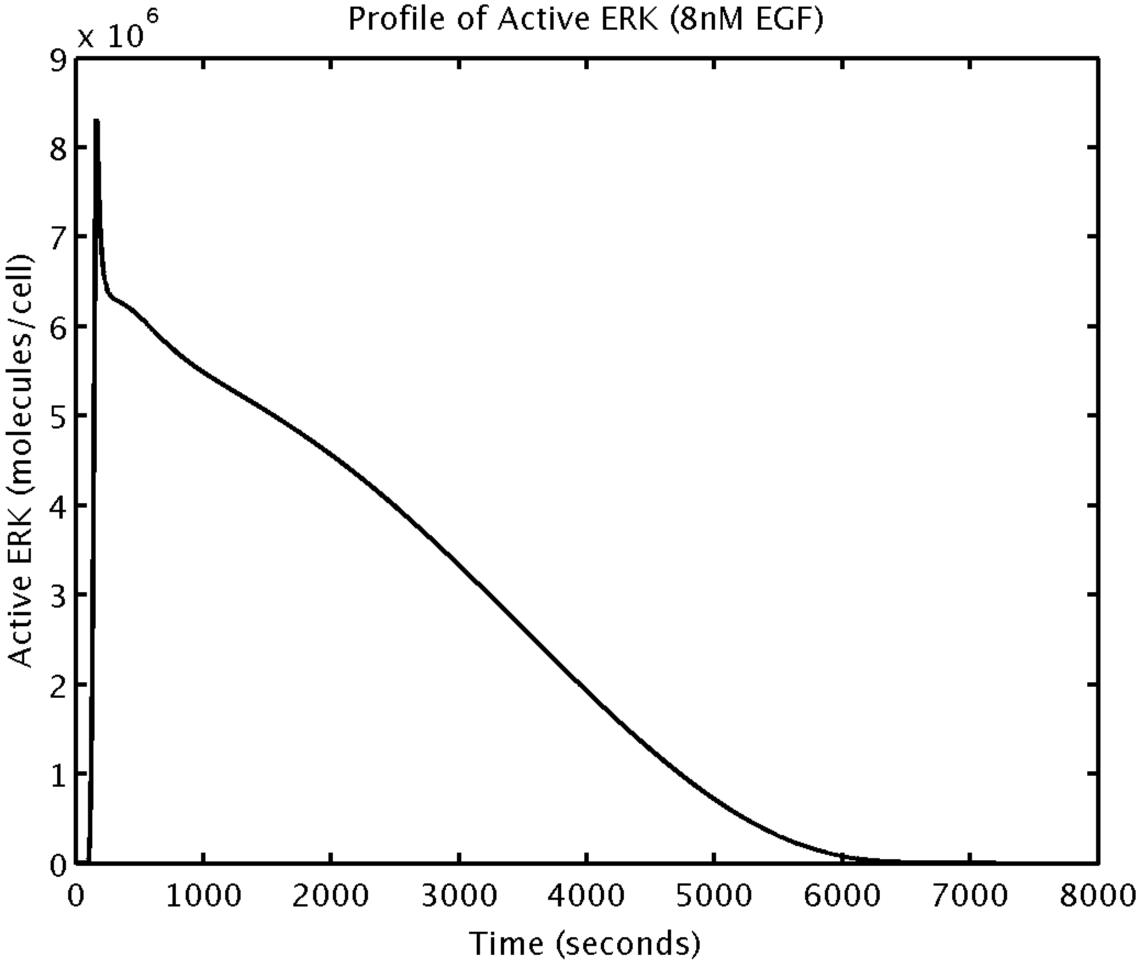

Supplementary Figure S2: The simulated time-dependent RasGTP activation dynamics of the drug-free EGFR-ERK pathway at 8nM EGF

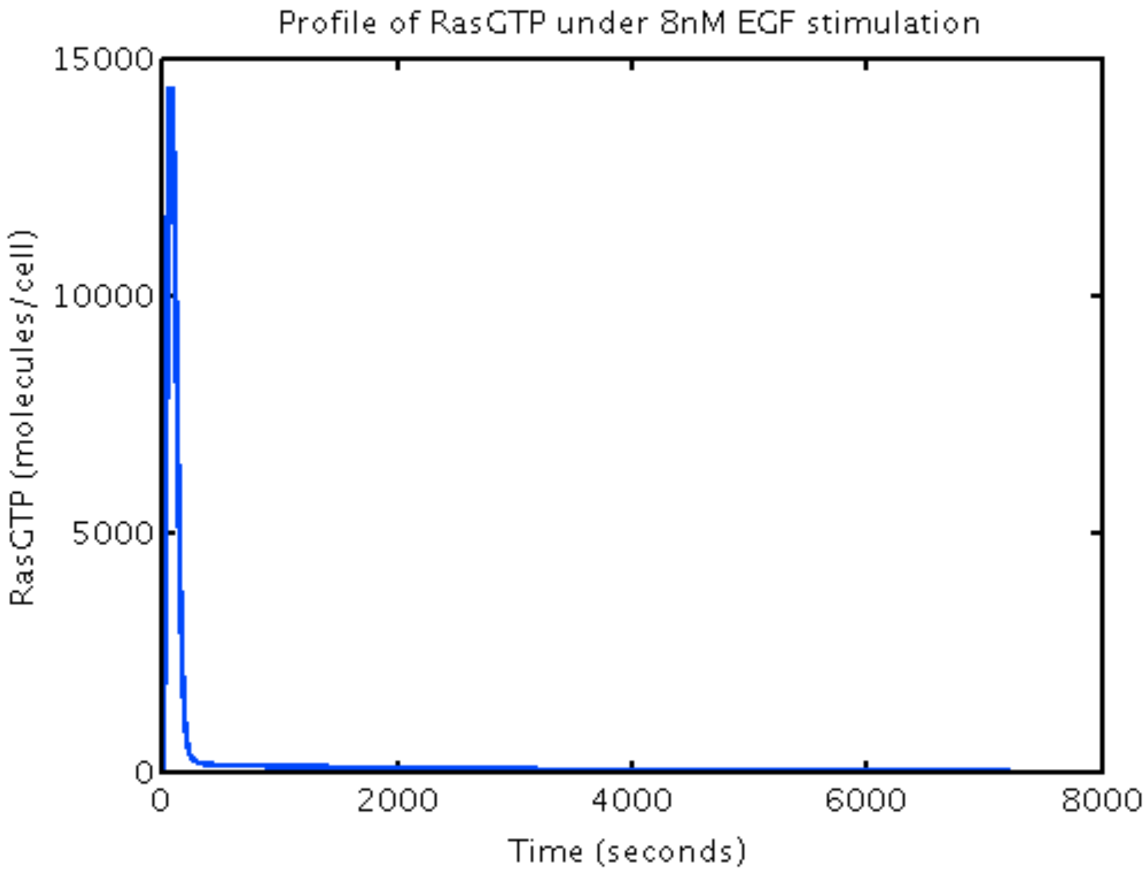

Supplementary Figure S3: The simulated time-dependent ERK activation dynamics of the drug-free EGFR-ERK pathway at 8nM EGF and different concentration of phosphatise 2 (PP2A)

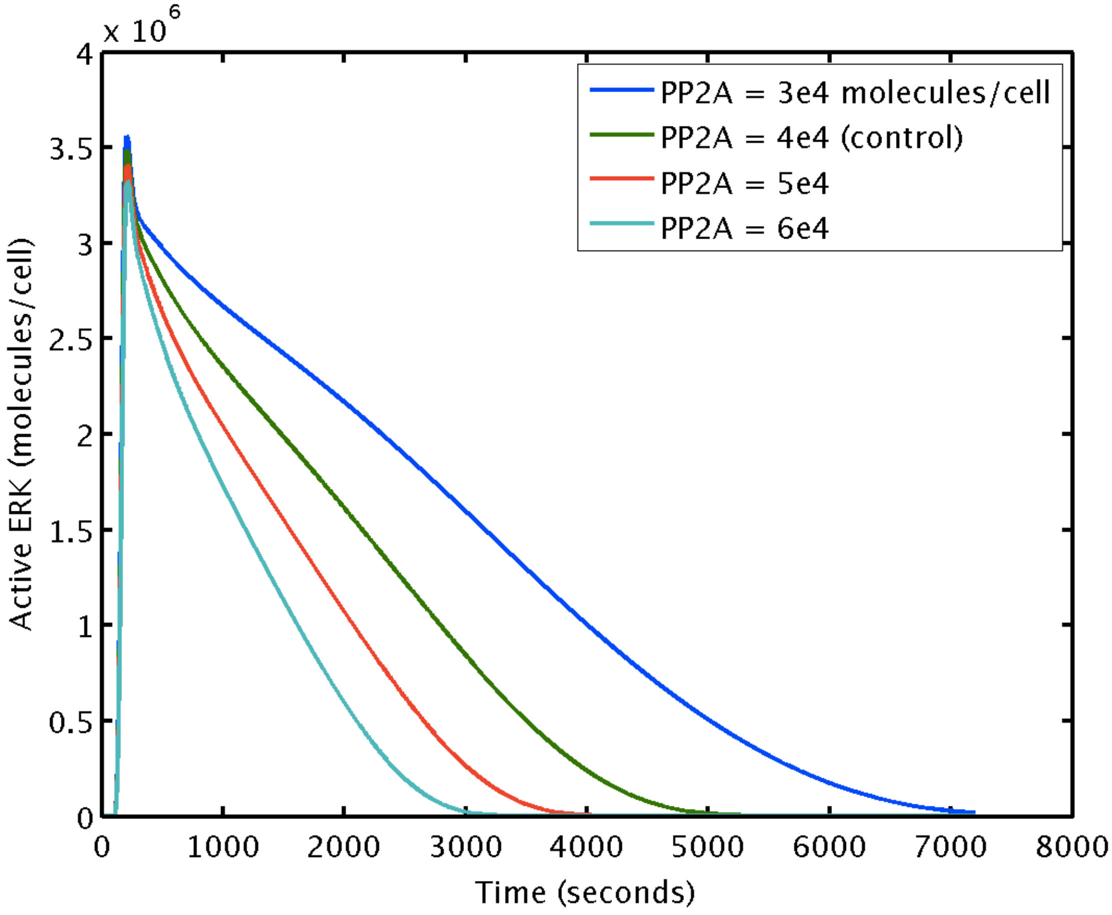

Supplementary Figure S4: The simulated time-dependent ERK activation dynamics of the drug-free EGFR-ERK pathway at 8nM EGF and different concentration of phosphatise 3 (MKP3)

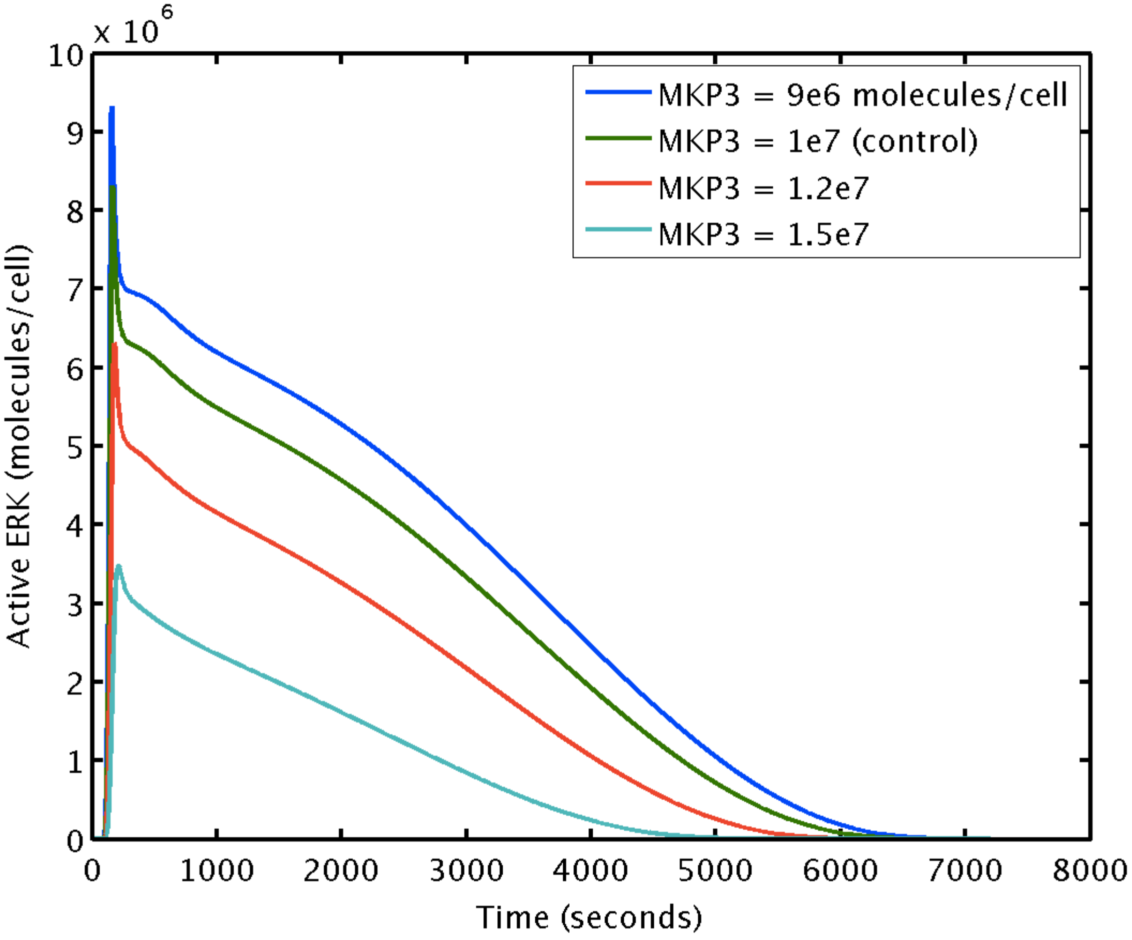

Supplementary Figure S5: The computed dose-effect curve of an EGFR inhibitor with 10nM IC50 value in inhibiting EGFR

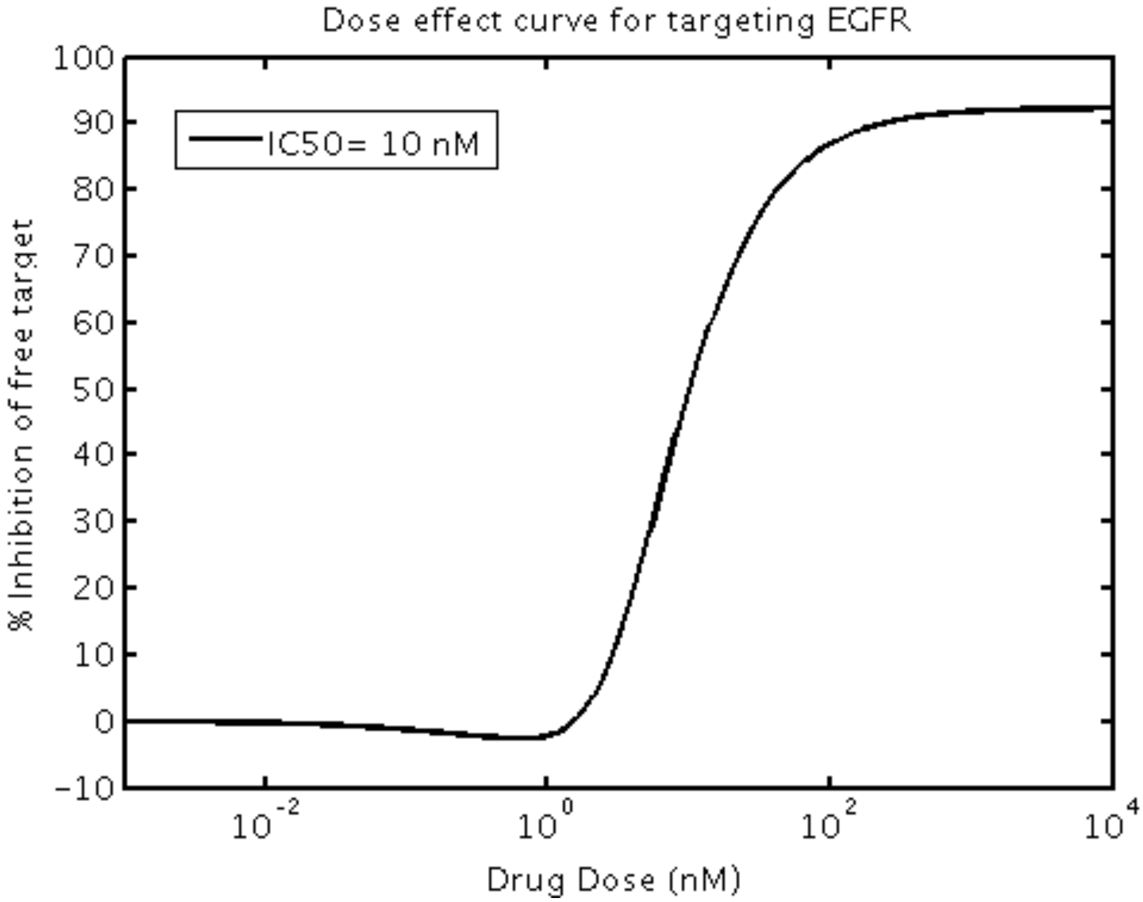

Supplementary Figure S6: The computed dose-effect curve of a BRaf inhibitor with 30nM IC50 value in inhibiting BRaf

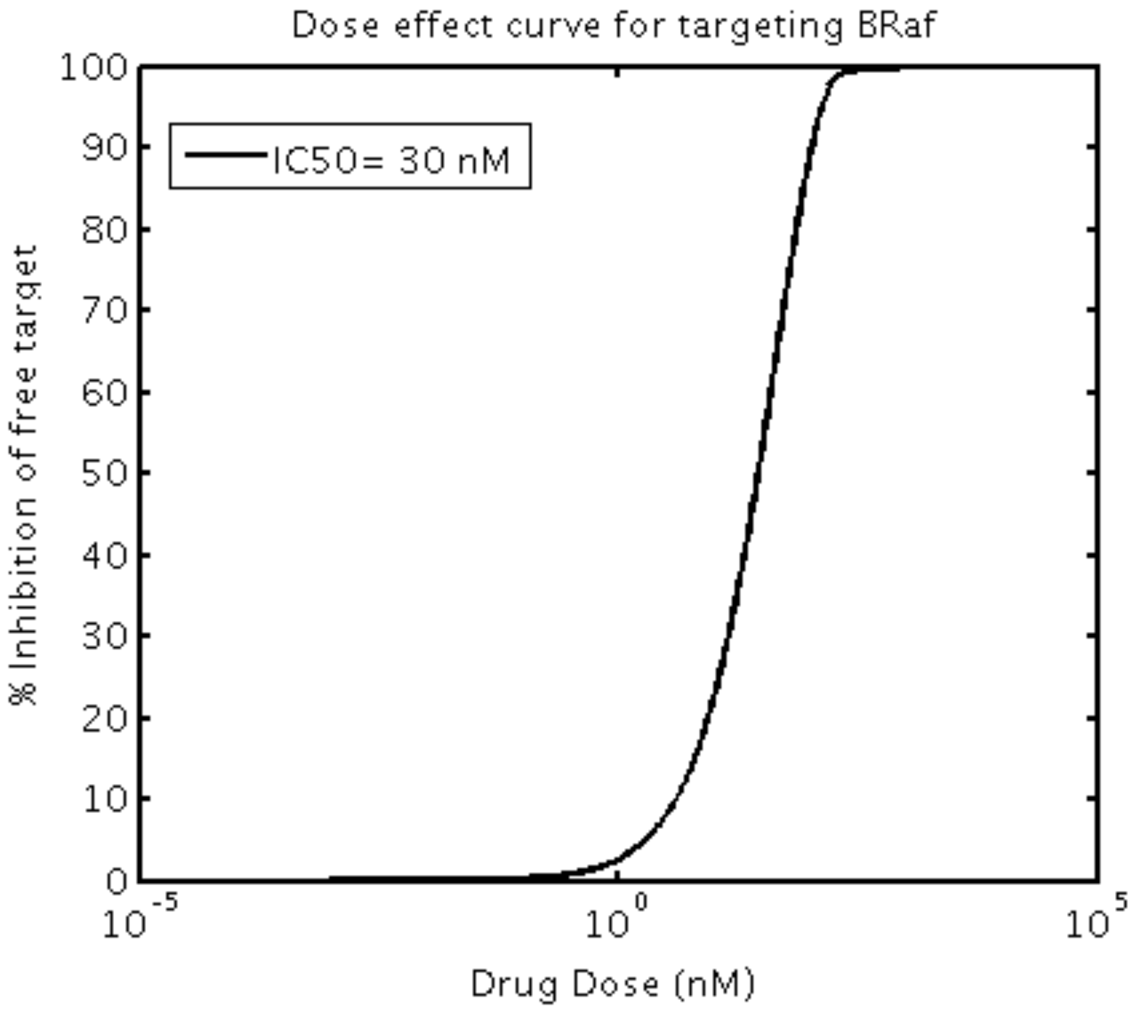

Supplementary Figure S7: The computed dose-effect curve of a Mek inhibitor with 15nM IC50 value in inhibiting Mek

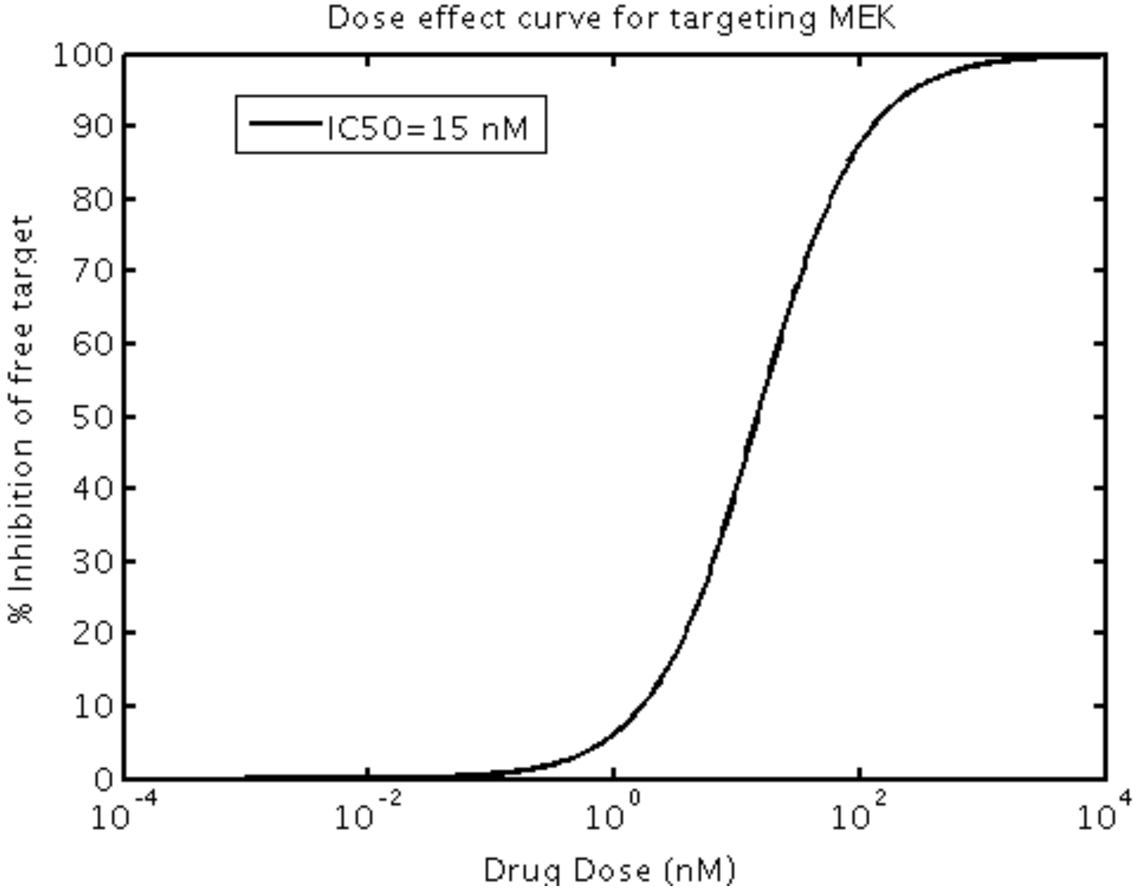

Supplement: Supplementary Tables and Figures [file srep40752-s1.pdf]
